# Characteristics and Clinical Predictors of *Chlamydia trachomatis* Infections Sustained by LGV Serovars Among Men Who Have Sex with Men

**DOI:** 10.3390/microorganisms14020262

**Published:** 2026-01-23

**Authors:** Alessia Siribelli, Angelo Roberto Raccagni, Sara Diotallevi, Riccardo Lolatto, Francesca Alberton, Emanuela Messina, Michela Sampaolo, Nicola Clementi, Roberto Burioni, Antonella Castagna, Silvia Nozza

**Affiliations:** 1 Infectious Diseases Unit, IRCCS San Raffaele Scientific Institute, 20132 Milan, Italy; 2 Infectious Diseases Unit, Vita-Salute San Raffaele University, 20132 Milan, Italy; 3 Laboratory of Microbiology and Virology, IRCCS San Raffaele Scientific Institute, 20132 Milan, Italy

**Keywords:** Lymphogranuloma venereum (LGV), *Chlamydia trachomatis* serovars, men who have sex with men (MSM), anorectal infection, predictors

## Abstract

This study aims to explore characteristics and clinical predictors of Lymphogranuloma venereum (LGV) and non-LGV *Chlamydia trachomatis* (Ct) serovars. We conducted a retrospective study on men who have sex with men (MSM) diagnosed with rectal or urethral Ct between 2015 and 2022 at the Infectious Diseases Unit of San Raffaele Scientific Institute, Milan, Italy. Nucleic acid amplification test with sequencing was used for Ct serovar determination. Individuals’ characteristics were described by median (interquartile, IQR) or frequency (%) and compared using Kruskal–Wallis or Chi-Square tests, as appropriate. Logistic regression model was used to identify predictors of LGV; multinomial logistic regression model, with LGV group as reference category, investigated factors associated with the LGV group (serovars L1, L2B, L2C), specific highly prevalent non-LGV serovars (D, E, G) or the non-amplifiable group. Overall, 211 MSM were included: 29.8% with LGV, 50.2% non-LGV and 19.9% non-amplifiable. Symptomatic cases were 46% of which 48% LGV; rectal infection was the most common (86%), followed by urethral (10%) and both sites (4%). People living with HIV were 91.5%; 31.3% had ≥1 concomitant STI and 65.4% ≥1 previous one. According to logistic regression analysis, after adjustment for the diagnosis of ≥1 concomitant and previous STI, LGV serovars were significantly associated with symptomatic infections (adjusted odds ratio, aOR = 6.05; 95%CI = 2.92, 13.13; *p* < 0.001) and anorectal site (aOR = 17.12; 95%CI = 3.17–319.17, *p* = 0.007) compared to non-LGV. Among MSM, almost 30% of Ct infections were due to LGV serovars. Presence of symptoms and anorectal site involvement, identified as clinical predictors of LGV, should guide clinicians during diagnosis.

## 1. Introduction

Globally, *Chlamydia trachomatis* (Ct) represents a leading cause of morbidity associated with bacterial sexually transmitted infections (STIs) [1]. Ct serovars L1-3 are responsible for Lymphogranuloma venereum (LGV), commonly affecting men who have sex with men (MSM), people living with HIV (PLWH) and HIV pre-exposure prophylaxis (PrEP) users [2].

Since the early 2000s, multiple European cities have reported sustained LGV spread with periodic surges, often linked to interconnected venue and overlapping risk clusters (e.g., multiple partners, group sex, chemsex). Across Europe, LGV notifications have risen steadily: in 2023, 22 countries reported a 41% increase over 2022 [2], with the Netherlands and Spain accounting for 77% of reports. LGV cases occurred predominantly among MSM and the proportion with HIV-negative status continued to rise (63% in 2023 among those with known status). In Italy, sentinel surveillance recorded a cumulative 303 LGV cases during 1991–2023, with a new peak in 2023 (46 cases of which 45 in MSM and 10 with HIV co-infection), representing a 3.5-fold increase compared to 2021 [2]. Molecular studies have shown that classical LGV serovar L2 is represented by several genovariants, including L2, L2b, L2c, L2f and additional L2b-related variants, with partially distinct geographical and population-specific distributions [3,4]. Data on European circulation of LGV serovars indicate a predominance by L2-related genovariants (L2, L2b and recombinant L2b/D) [5]. Particularly in Southern European countries, L2b has been repeatedly reported as the predominant genovariant among MSM, although other variants such as L2c and L2f have also been detected [6,7].

However, data on the prevalence of individual L2 genovariants in Europe remain limited. Clarifying which L2 genovariants are circulating in specific settings, along with their associated clinical manifestations, is therefore crucial to complete and enrich the epidemiological picture of this geographical area, thereby supporting more targeted surveillance.

The natural history of LGV consists of a primary mucosal lesion, regional lymphadenitis and late fibrotic complications. Nowadays, the most common clinical phenotype is dominated by proctitis/proctocolitis with rectal pain, bleeding, discharge, constipation, tenesmus and systemic symptoms [8]. Importantly, a portion of rectal L-serovar infections is asymptomatic, creating a surveillance gap and raising the question of whether all L-serovar detections should be labeled “LGV disease” or reserved for those with associated clinical criteria.

Risk is defined by behaviors more than by demographics alone. Receptive anal intercourse without condoms, higher partner numbers and recent or recurrent STIs are repeatedly associated with LGV [2]. Both PWH and PrEP users are frequently represented in clinic-based cohorts because of testing access and behavioral profiles, although HIV status per se is not thought to be a biologic driver of LGV risk after adjusting for behavior [9].

From a diagnostic perspective, standard NAAT platforms detect Ct with high analytical sensitivity, but they do not differentiate LGV from non-LGV [10]. Reflex testing to distinguish L-serovars can be used in case of positive rectal NAATs to guide management. Single-copy targets may fail in low-load specimens, contributing to non-amplifiable results, while dual-target strategies and plasmid-directed assays can mitigate this problem and shorten the interval to a definitive LGV vs. non-LGV result [11,12].

Beyond epidemiology, genotyping often supports, in clinical practice, treatment management. Indeed, therapeutic pathways diverge according to genotype and phenotype: guidelines recommend doxycycline 100 mg twice daily for 21 days for confirmed or strongly suspected LGV, combined with partner notification and evaluation for co-STIs (13). For non-LGV rectal Ct, 7 days of doxycycline is preferred [13]. In contrast, several recent observational studies indicate that selected asymptomatic or clinically mild rectal LGV can be effectively managed with shorter treatment durations [14,15,16]. Nonetheless, in the absence of randomized trials, most authorities continue to endorse the 21-day regimen for LGV (8, 13) while research on de-escalation proceeds.

Against this background, we designed our study with two main objectives. First, we aimed to describe the distribution of LGV and non-LGV serovars, with particular attention to circulating L2 genovariants, comparing these patterns with those previously reported in Italian cohorts. Second, we sought to explore the clinical characteristics and predictors associated with LGV versus non-LGV infections in this population.

Within this landscape, serovar determination remains pivotal, as treatment duration and partner management differ for LGV versus non-LGV. However, heterogeneity in access to genotyping limits its real-time utility in many clinics. We therefore evaluated whether readily obtainable, low-cost clinical indicators—principally symptomatology and anatomical site of infection—exhibit sufficient discriminative capacity to guide empiric management at presentation when rapid typing is unavailable.

## 2. Materials and Methods

This retrospective study evaluated MSM followed at the Infectious Diseases Unit of San Raffaele Scientific Institute in Milan as outpatients to receive HIV treatment or PrEP. People diagnosed, between 2015 and 2022, with Ct infection, detected by NAAT on rectal or urethral swabs with Cobas CT/NG test on the Cobas 6800 system (Roche, Basel, Switzerland), were included. All positive samples were tested for serovars determination sequencing to identify specific Ct genotype, regardless of the presence or absence of symptoms. LGV positive samples were amplified by nested Omp1 PCR with primers previously described [17]. In the details, Omp1 gene was sequenced for both genovar determination and discrimination amongst genotypes. Agarose gel electrophoresis of the PCR products was performed for checking amplicons (624 bp) and acceptable purity. Then, Sanger sequencing was performed using the BigDye Terminator v3.1 Cycle Sequencing Kit (Life Technologies, Carlsbad, CA, USA) with the same PCR-inner primers. Sequencing was performed by using ABI 3730xl capillary (Applied Biosystems, Foster City, CA, USA) sequencer with ABI POP-7 Polymer (Applied Biosystems, Foster City, CA, USA). Electropherograms were analyzed with SeqScape 3 software v3.0 and the consensus sequence was compared with known Ct LGV strains by using the BLAST search tool (22.01.2026) to identify specific serovars. Finally, a phylogenetic tree analysis was carried out with ClustalX software to confirm specific genotypes by including different genotypes of Ct and other species of Chlamydia as an outgroup (Supplementary Materials). Sequences analysis did not detect any SNPs that suggest new L2 variants.

Consequently, the population was divided in LGV and non-LGV groups, the latter further classified in specific subgroups, according to non-LGV (D-J) serovars. Some samples were not sequenced, due to non-amplifiable results, and therefore assembled in a separate group.

Clinical data on symptoms were collected from medical records for the entire population. Symptomatic Ct infection was defined as the presence of at least one rectal or urethral symptom reported at the time of diagnosis, including anal pain, anal discharge, rectal bleeding, tenesmus, constipation, diarrhea, dysuria, and urethral discharge. In the absence of these symptoms, infections were classified as asymptomatic. All microbiologically confirmed cases of Ct infections were treated according to the current Centers for Diseases Control and Prevention (CDC) guidelines.

Individual characteristics at baseline, defined as date of *Chlamydia trachomatis* diagnosis, were reported: qualitative variables as absolute values and frequency (%) and quantitative variables by median and interquartile range (IQR). These variables were compared using Kruskal–Wallis or Chi-Square tests, as appropriate. Binary logistic regression model was used to identify predictors of LGV vs. non-LGV, excluding non-amplifiable genotypes. Multinomial logistic regression model was used to evaluate associations with specific genotypes (D, E, G and non-amplifiable), with genotype L as the reference category. In both models, the following covariates were included because they were considered clinically relevant: site of infection (anal versus non-anal), presence of symptoms at diagnosis (yes versus no), diagnosis of at least one concomitant STI and history of at least one previous STI. Results are reported as adjusted odds ratios (aOR) with 95% confidence intervals (Cis) and *p*-values. All statistical tests were two-sided at 5% level and were carried out using R Statistical Software, version 4.2.2 (R Foundation for Statistical Computing, Vienna, Austria). Clinical data, individuals’ characteristics and microbiologic results were retrieved from the Centro San Luigi (CSL) HIV Cohort electronic health records. Recorded data are anonymized and managed according to the Good Clinical Practice Guidelines published by the World Medical Association Declaration of Helsinki.

## 3. Results

Overall, 211 MSM were included: at baseline, median age was 39.7 [IQR: 34.1–49.3] years; 201 (95.3%) were Caucasian, 9 (4.3%) Hispanic, and 1 (0.5%) Asian. People living with HIV were 193 (91.5%). A total of 98 (46.4%) were symptomatic; 66 (31.3%) and 138 (65.4%) had at least one concomitant or previous STI, respectively. Rectal site was the most common infection site with 181 (86.2%) positive swabs, followed by urethral with 21 (10.0%) and both anal-urethral with 6 (2.86%). Sequencing results of Ct were as follows: 63 (29.8%) were LGV, 106 (50.2%) non-LGV and 42 (19.9%) non-amplifiable. LGV serovars were distributed as follows: 3 (4.7%) L1, 27 (42.8%) L2, 11 (17.4%) L2b and 22 (34.9%) L2c; non-LGV were 42 (39.6%) D, 29 (27.3%) E, 2 (1.8%) F, 24 (22.6%) G, 1 (0.9%) IA, 7 (6.6%) J, and 1 (0.9%) J2. At diagnosis, symptoms were present in 48 (76.2%) of LGV, compared with 40 (37.7%) non-LGV and 10 (23.8%) non-amplifiable cases, with significant difference between groups (*p* <0.001). Rectal site was the most common in LGV [60 (95.2%)], non-LGV [87 (82.1%)] and non-amplifiable [34 (82.9%)]. Diagnosis of at least one concomitant or previous STI was observed, respectively, in 25 (39.7%) and 45 (71.4%) of LGV, 29 (27.4%) and 65 (61.3%) of non-LGV, and 12 (28.6%) and 28 (66.7%) of non-amplifiable, without significant differences between groups.

Overall, the most common concomitant and previous STIs were, respectively, gonorrhea [34 (16.1%)] and syphilis [73 (34.6%)]. As for LGV group, mycoplasma [15 (23.8%)] was most frequently diagnosed as concomitant infection, while syphilis [26 (41.3%)] was diagnosed as previously described. In the non-LGV group, the most common concomitant and previous STIs were, respectively, gonorrhea [18 (17.0%)] and syphilis [34 (32.1%)]. Significant differences between groups were found regarding concomitant mycoplasma (*p* < 0.001) and ureaplasma (*p* = 0.001) infections, being more frequent in LGV cases. Summary of demographic and clinical characteristics of LGV vs. non-LGV vs. non amplifiable are displayed in Table 1.

Summary of characteristics according to specific LGV serovars are summarized in Table 2: no significant differences between subgroups were found, except for presence of symptoms and diagnosis of at least one concomitant STIs, being more frequent in L2 compared to other L-serovars.

In the logistic regression analysis neither LGV nor non-LGV serovars were significantly associated with diagnosis of at least one concomitant and previous STI. LGV serovars were significantly associated with symptomatic infections [adjusted odds ratio (aOR): 6.05; 95%CI: 2.92, 13.13; *p* < 0.001)] and anorectal site involvement (aOR: 17.12; 95%CI: 3.17, 319.17, *p* = 0.007) compared to non-LGV.

In the multinomial logistic regression model, symptomatic infections were less frequently associated with specific highly prevalent non-LGV serovars, including genotype (Gt) D (aOR: 0.28; 95%CI: 0.12, 0.67; *p* < 0.001), Gt E (aOR: 0.10; 95%CI: 0.04, 0.31; *p* < 0.001), Gt G (aOR: 0.12; 95%CI: 0.04, 0.36; *p* < 0.001), and non-amplifiable group (aOR: 0.09; 95%CI: 0.03, 0.23; *p* < 0.001) compared to LGV serovars (Table 3). Anorectal site infection was less likely associated with Gt D (aOR: 0.09; 95%CI: 0.01, 0.86; *p* = 0.04), Gt E (aOR: 0.03; 95%CI: 0.00, 0.26; *p* = 0.002), Gt G (aOR: 0.05; 95%CI: 0.00, 0.48; *p* = 0.01) and non-amplifiable group (aOR: 0.06; 95%CI: 0.01, 0.54; *p* = 0.01) compared to LGV serovars.

## 4. Discussion

In this cohort of MSM, the majority of Ct infections were non-LGV, in agreement with similar data described in the literature [18,19]. LGV cases accounted for almost 30% of all Ct infections, in line with a previous study conducted in Italy [7]. Particularly, in the Bologna cohort described by Marangoni et al. [7], the most frequently detected L2 genovariants among MSM were L2f, L2b, and L2-L2b/D-Da, together with other L2b variants. In our setting, we identified a predominance of L2 (42.8%), L2b (17.4%) and L2c (34.9%), suggesting that comparable strains are circulating across Italian MSM networks, with a relative increase in L2c over the study period compared to the Bologna cohort.

A higher number of LGV genotype infections were symptomatic compared to non-LGV. Asymptomatic LGV genotype infections constituted about 24% of all LGV cases, similarly to data previously described in a cohort of MSM from the United Kingdom [20]. Our findings reinforce a pragmatic clinical approach: in MSM presenting with urogenital and anorectal symptoms, LGV should be strongly suspected and empiric doxycycline initiated when immediate typing is unavailable, alongside partner notification. Conversely, in asymptomatic screen-detected rectal Ct, early differentiation of LGV vs. non-LGV may still have a role for treatment duration and follow-up [21]. On the other hand, a current debate questions whether asymptomatic infections due to L-serovars should be referred to as LGV. An editorial by Handsfield [22] states that LGV infections could include only clinical cases, while asymptomatic infections should be regarded as L-serovars infections.

Interestingly, a proportion of LGV infections in our cohort were asymptomatic, including cases caused by L2b genovariants. This observation aligns with previous reports indicating that even highly invasive LGV strains can occasionally present without overt proctitis, potentially reflecting early detection, partial treatment, host immune factors, or co-circulation of strains with varying pathogenicity [18,20]. Among non-LGV serovars, serovar D, which harbors a cytolytic toxin gene, has been associated with more pronounced epithelial damage in experimental models [23,24]; however, in our clinical cohort, serovar D infections showed a heterogeneous spectrum of symptoms, ranging from asymptomatic carriage to overt proctitis. L2c, described as a recombinant L2/D strain with a functional toxin gene [25], was detected only in a small number of cases, which limited our ability to draw firm conclusions about its clinical behavior. Nevertheless, the presence of asymptomatic LGV and non-LGV infections across different serovars underscores the need for routine screening in high-risk MSM, regardless of symptoms.

Rectal Ct was more common than urethral and urethral/rectal in both LGV and non-LGV. The reason may vary depending on the population, although some data suggest that these findings may be due to behavioral more than demographic characteristics [26].

Previous and concomitant STIs were frequently registered both in LGV and non-LGV: gonorrhea, mycoplasma and ureaplasma being the most common concomitant infections, whereas syphilis was the most common previous infection in both groups. No statistical difference between groups was found regarding diagnosis of at least one concomitant or previous STIs. However, concomitant infection with mycoplasma and ureaplasma was significantly more common in LGV compared to non-LGV. These data are in discordance with previous studies [27,28] reporting higher rates of concomitant STIs, particularly syphilis, associated with LGV. The specific data for mycoplasma/ureaplasma coinfections deserves cautious interpretation. Ureaplasma detection at rectal or urogenital sites may represent colonization rather than pathogenic infection [29]. Conversely, *Mycoplasma genitalium* is more often implicated in proctitis [30] and may cluster in the same sexual networks that sustain LGV transmission. Thus, a higher prevalence alongside LGV could reflect shared behavioral networks rather than direct biological interaction [31].

Comparison between specific L-serovars showed that presence of symptoms and diagnosis of at least one STI was more frequent in L2, with statistical difference between subgroups. This pattern may reflect several, although not mutually exclusive, mechanisms. First, it is biologically plausible that L2 lineages exhibit greater rectal tissue tropism and/or higher organism burden [5], thereby increasing the likelihood of symptomatic proctitis, of being tested for, and diagnosed with, concomitant STIs. Second, the sexual-network context in which L2 circulates may involve higher partner concurrency [32] or chemsex use [33], features that are themselves associated with a greater background prevalence of STIs. In this scenario, the observed association would arise from network-level confounding rather than intrinsic virulence. Third, symptomatic individuals are more likely to undergo three-site NAAT and broader multiplex testing, inflating detection of coinfections in the L2 stratum if L2 is over-represented among symptomatic cases.

Neither concomitant nor previous STIs confirmed to be associated with LGV serovars infections. These findings are in contrast with those of a retrospective study [34] conducted in an outpatient clinic in the Netherlands, where they observed that prior STIs were significantly predictive of LGV infections. Moreover, a study by Pathela et al. [35] showed that history of syphilis was an independent risk factor of LGV. Foschi et al. [27] found HIV and syphilis to be stronger predictors of LGV infection, compared to other STIs.

In our cohort, predictors of LGV were presence of symptoms and rectal site infection, in both logistic regression analysis. Previous studies [34,35,36], comparing characteristics and predictors of LGV vs. non-LGV infections, showed that anorectal symptoms were significantly predictive of LGV. Pallawela et al. [37] observed that anal discharge, tenesmus, constipation and weight loss were commonly associated with LGV infections, with only tenesmus and constipation as statistically significant predictors of LGV infections in the multivariate analysis.

There are limitations in this study. Due to the retrospective design, sexual behaviors were not included as possible risk factors since this data is not systematically collected during follow-up visits. Since our cohort consists entirely of MSM, with median age being 40 years, interpretation of data should be limited to this specific population. Moreover, in almost 20% of cases, a specific serovar could not be identified, due to non-amplifiable results. The proportion of non-amplifiable genotyping results observed in this study is consistent with drop-out on single-copy targets in low-bacterial-load specimens [12]. Incorporating dual-target approaches, including plasmid-oriented assays, can reduce indeterminate outcomes and accelerate LGV/non-LGV triage [11]. When resources permit, higher-resolution genotyping can support micro-cluster detection and outbreak management without delaying initial care.

Nonetheless, this study compares clinical and epidemiological characteristics of LGV and non-LGV serovar infections, offering insights into possible predictors which could guide clinicians through the diagnostic process.

## 5. Conclusions

Among MSM attending specialist care, LGV serovars accounted for roughly one-third of all Ct infections. Prior or concomitant STIs were common in both LGV and non-LGV groups, yet we found no statistically significant difference in their overall prevalence between groups. By contrast, two readily ascertainable clinical features—presence of symptoms and anorectal site of infection—emerged as the only independent predictors of LGV. Taken together, these findings underscore that careful clinical surveillance remains pivotal for early diagnostic orientation and, consequently, for selecting the appropriate therapeutic pathway. Finally, important evidence gaps remain. The absence of a strong association between LGV and other STIs in our cohort, despite signals in the prior literature, highlights the need for prospective multicenter studies incorporating standardized three-site testing, time-updated behavioral evaluation and rapid LGV discrimination at baseline. In parallel, randomized trials are warranted to determine whether shorter doxycycline courses are non-inferior for non-severe rectal LGV. Addressing these priorities will refine the diagnostic approach and optimize treatment strategies for MSM at risk of Ct infection.

## Figures and Tables

**Table 1:** Demographic and clinical characteristics of LGV, non-LGV and non-amplifiable group.

	LGV	Non-LGV	Non-Amplifiable	*p*
	*N* = 63	*N* = 106	*N* = 42	
**Male sex**	63 (100%)	106 (100%)	42 (100%)	-
**Age**	39.7 [33.6;48.2]	41.2 [34.7;50.1]	39.1 [34.9;48.3]	0.888
**Risk factors:**				1.000
*Ex-drug users*	0 (0.00%)	1 (0.94%)	0 (0.00%)	
*MSM*	63 (100%)	105 (99.1%)	42 (100%)	
**HIV at baseline**	57 (90.5%)	98 (92.5%)	38 (90.5%)	0.848
**Sites of infection:**				0.066
*Anal*	60 (95.2%)	87 (82.1%)	34 (82.9%)	
*Anal and Urethral*	2 (3.17%)	3 (2.83%)	1 (2.44%)	
*Pharyngeal*	0 (0.00%)	2 (1.89%)	0 (0.00%)	
*Urethral*	1 (1.59%)	14 (13.2%)	6 (14.6%)	
**Presence of symptoms**	48 (76.2%)	40 (37.7%)	10 (23.8%)	<0.001
**≥1 concomitant STI**	25 (39.7%)	29 (27.4%)	12 (28.6%)	0.226
*Gonorrhea concomitant*	9 (14.3%)	18 (17.0%)	7 (16.7%)	0.894
*HSV concomitant*	0 (0.00%)	1 (0.94%)	1 (2.38%)	0.447
*Syphilis concomitant*	1 (1.59%)	2 (1.89%)	2 (4.76%)	0.501
*Mycoplasma concomitant*	15 (23.8%)	5 (4.72%)	2 (4.76%)	<0.001
*Mpox concomitant*	1 (1.59%)	3 (2.83%)	2 (4.76%)	0.746
*Ureaplasma concomitant*	14 (22.2%)	6 (5.66%)	1 (2.38%)	0.001
**≥1 previous STI**	45 (71.4%)	65 (61.3%)	28 (66.7%)	0.402
*Gonorrhea previous*	17 (27.0%)	21 (19.8%)	12 (28.6%)	0.404
*HSV previous*	3 (4.76%)	11 (10.4%)	3 (7.14%)	0.444
*Syphilis previous*	26 (41.3%)	34 (32.1%)	13 (31.0%)	0.410
*Mycoplasma previous*	10 (15.9%)	17 (16.0%)	10 (23.8%)	0.490
*Mpox previous*	0 (0.00%)	2 (1.89%)	1 (2.38%)	0.591
*Ureaplasma previous*	15 (23.8%)	16 (15.1%)	9 (21.4%)	0.339
*Chlamydia previous*	15 (23.8%)	26 (24.5%)	11 (26.2%)	0.962

LGV: Lymphogranuloma venereum; MSM: men who have sex with men; STI: sexually transmitted infection; HSV: herpes simplex virus. Statistical tests used: Chi-square test for categorical variable; Kruskal–Wallis test for continuous variables.

**Table 2:** Summary of demographic and clinical characteristics according to L-serovars.

	L1	L2	L2b	L2c	*p*
	*N* = 3	*N* = 27	*N* = 11	*N* = 22	
**Age**	31.2 [28.1;36.2]	39.3 [33.4;47.0]	43.5 [35.5;55.3]	40.9 [33.5;46.8]	0.265
**HIV at baseline**	3 (100%)	26 (96.3%)	10 (90.9%)	18 (81.8%)	0.386
**HCV Ab at baseline**	1 (33.3%)	4 (14.8%)	0 (0.00%)	3 (13.6%)	0.628
**HBsAg at baseline**	0 (0.00%)	0 (0.00%)	1 (9.09%)	1 (4.55%)	0.141
**Ethnicity:**					0.354
*Caucasian*	2 (66.7%)	25 (92.6%)	11 (100%)	1 (4.5%)	
*Other*	1 (33.3%)	2 (7.41%)	0 (0.00%)	21 (95.5%)	
**Sites of infection**					0.207
*Anal*	3 (100%)	27 (100%)	10 (90.9%)	20 (90.9%)	
*Anal and Urethral*	0 (0.00%)	0 (0.00%)	0 (0.00%)	2 (9.09%)	
*Urethral*	0 (0.00%)	0 (0.00%)	1 (9.09%)	0 (0.00%)	
**Presence of symptoms:**	1 (33.3%)	24 (88.9%)	9 (81.8%)	14 (63.6%)	0.047
**Concomitant infections:**					
*Gonorrhea*	0 (0.00%)	6 (22.2%)	1 (9.09%)	2 (9.09%)	0.653
*Syphilis*	0 (0.00%)	0 (0.00%)	0 (0.00%)	1 (4.55%)	0.571
*Mycoplasma*	0 (0.00%)	9 (33.3%)	2 (18.2%)	4 (18.2%)	0.511
*Mpox*	0 (0.00%)	1 (3.70%)	0 (0.00%)	0 (0.00%)	1.000
*Ureaplasma*	0 (0.00%)	10 (37.0%)	1 (9.09%)	3 (13.6%)	0.123
**≥1 concomitant STI:**	0 (0.00%)	16 (59.3%)	3 (27.3%)	6 (27.3%)	0.037
**Previous STIs:**					0.811
*Gonorrhea*	1 (33.3%)	7 (25.9%)	4 (36.4%)	5 (22.7%)	0.811
*HSV*	0 (0.00%)	1 (3.70%)	1 (9.09%)	1 (4.55%)	0.806
*Syphilis*	1 (33.3%)	9 (33.3%)	5 (45.5%)	11 (50.0%)	0.694
*Mycoplasma*	0 (0.00%)	3 (11.1%)	4 (36.4%)	3 (13.6%)	0.282
*Ureaplasma*	0 (0.00%)	8 (29.6%)	4 (36.4%)	3 (13.6%)	0.344
*Chlamydia*	1 (33.3%)	4 (14.8%)	2 (18.2%)	8 (36.4%)	0.268
**≥1 STI previous**	2 (66.7%)	17 (63.0%)	9 (81.8%)	17 (77.3%)	0.613

HCV Ab: hepatitis C virus antibodies; HBsAg: HBV surface antigen; STI: sexually transmitted infection; HSV: herpes simplex virus.

**Table 3:** Multinomial logistic regression table.

*Variables*	Category	Genotype D §		Genotype E §		Genotype G §		Genotype Not Amplifiable §	
		Adjusted OR (95%CI)	*p*	Adjusted OR (95%CI)	*p*	Adjusted OR (95%CI)	*p*	Adjusted OR (95%CI)	*p*
**Symptoms**	Yes vs. no	0.28 (0.12, 0.67)	<0.001	0.10 (0.04, 0.31)	<0.001	0.12 (0.04, 0.36)	<0.001	0.09 (0.03, 0.23)	<0.001
**Sites**	Rectal vs. non-rectal	0.09 (0.01, 0.86)	0.04	0.03 (0.00, 0.26)	0.002	0.05 (0.00, 0.48)	0.01	0.06 (0.01, 0.54)	0.01
**≥1 concomitant STI**	Yes vs. No	0.72 (0.30, 1.73)	0.46	0.85 (0.29, 2.51)	0.76	0.37 (0.11, 1.33)	0.13	0.75 (0.29, 1.95)	0.56
**≥1 previous STI**	Yes vs. No	1.44 (0.56, 3.68)	0.44	0.43 (0.15, 1.20)	0.11	0.87 (0.30, 2.56)	0.80	1.26 (0.48, 3.31)	0.64

§ The reference category is Genotype L (L1, L2, L2b, L2c).

## Data Availability

The data presented in this study are available on request from the corresponding author due to privacy restrictions.

## Supplementary Materials

The following supporting information can be downloaded at https://www.mdpi.com/article/10.3390/microorganisms14020262/s1, Figure S1: Reference tree for patient genotype identification: phylogram; Figure S2: Reference tree for patient genotype identification: cladogram.
