# Supplementary material for: Characteristics and Clinical Predictors of Chlamydia trachomatis Infections Sustained by LGV Serovars Among Men Who Have Sex with Men
Source: Microorganisms. 2026 Jan 23;14(2):262. doi: 10.3390/microorganisms14020262 (PMC12943210; doi:10.3390/microorganisms14020262)

Supplementary Figure S1. Reference tree for patient genotype identification: phylogram.

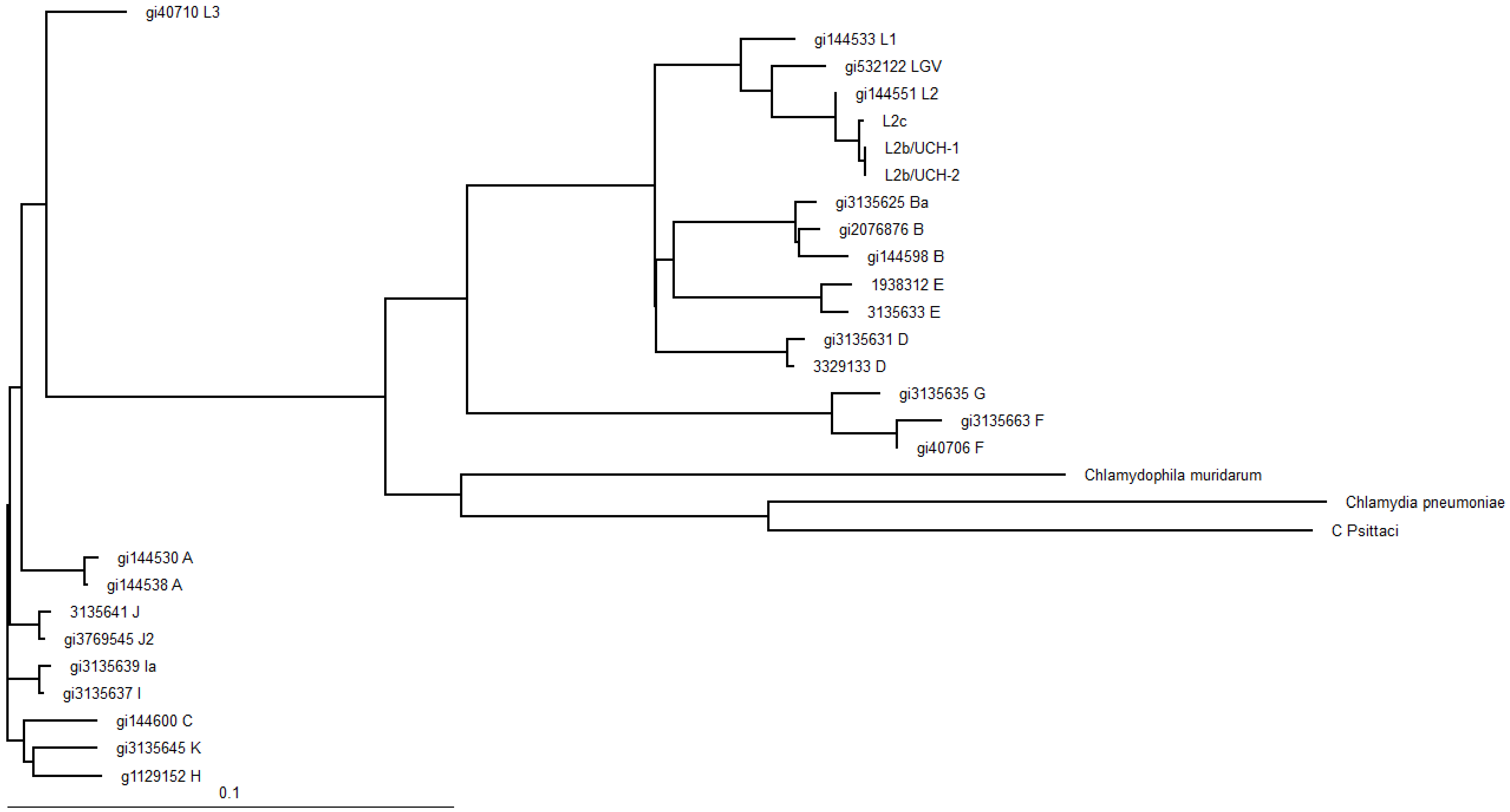

Supplementary Figure S2. Reference tree for patient genotype identification: cladogram.

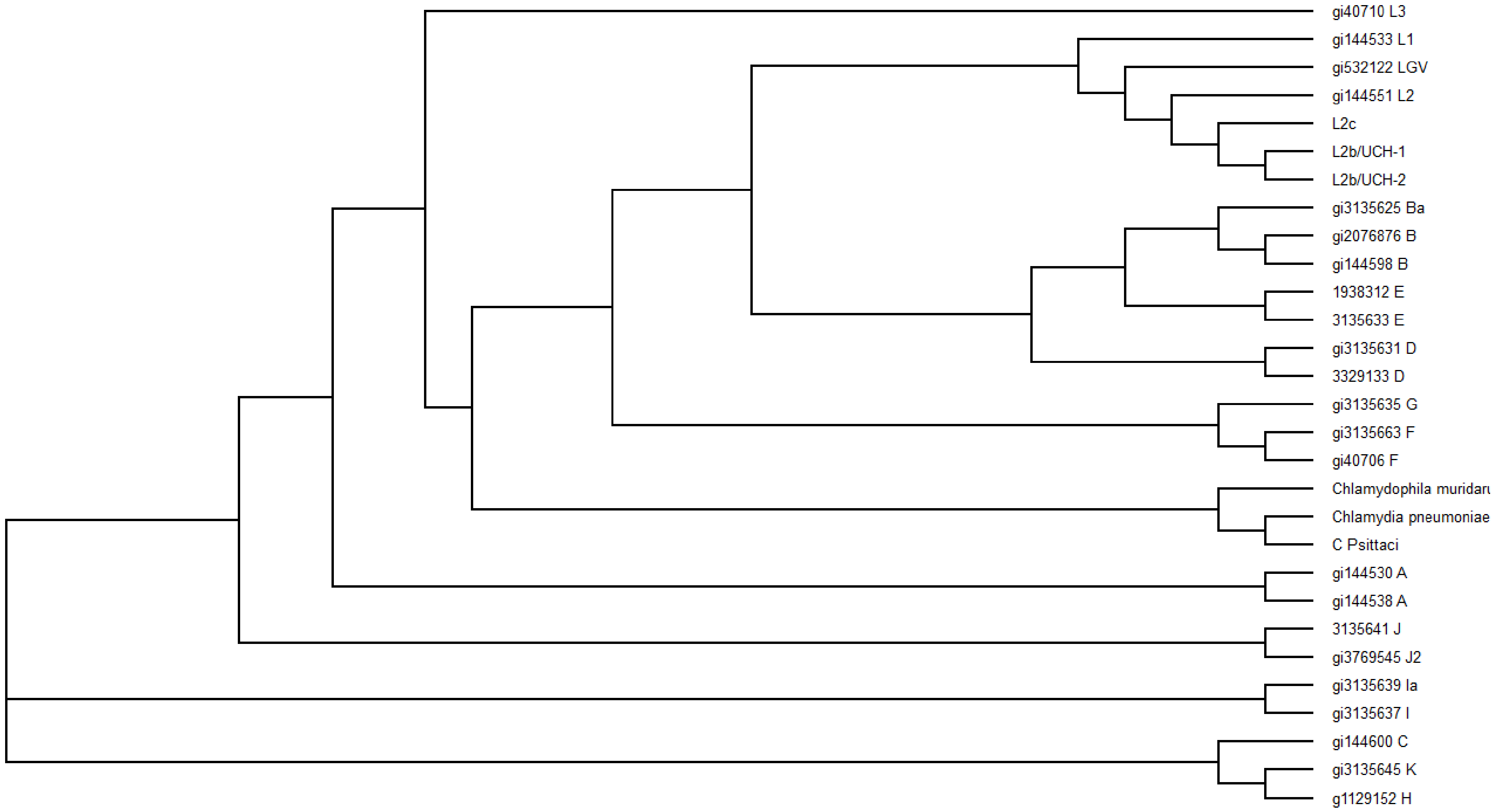

Supplement: Supplementary file 1 [file microorganisms-14-00262-s001.zip › microorganisms-4007586-supplementary.pdf]
